# Supplementary material for: Beyond the perception of present economic inequality: How people construe past, present and future wealth gaps
Source: PLoS One. 2026 Jan 9;21(1):e0340320. doi: 10.1371/journal.pone.0340320 (PMC12788628; doi:10.1371/journal.pone.0340320)
Supplement: S1 File — This file contains all Supporting Information figures, tables, and additional analyses not included in the manuscript. (DOCX) [file pone.0340320.s001.docx]

## *S1 Fig. Google Trends’ results for the keyword “economic inequality” between 2006 and 2024
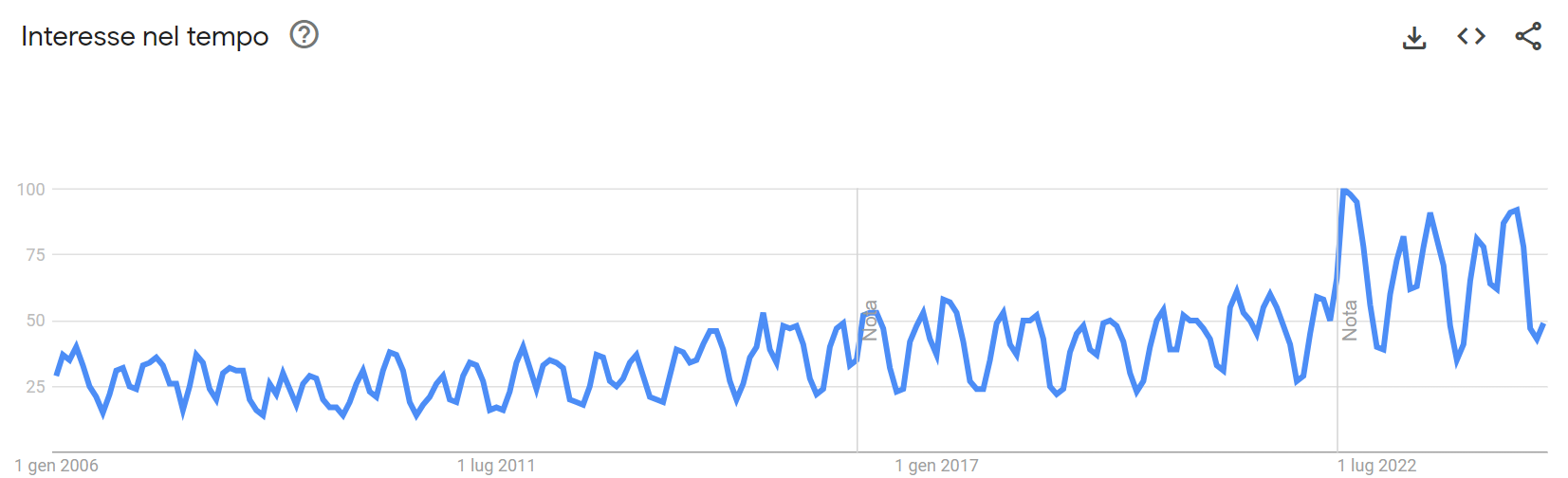
*

**A Methodological Note.** As discussed in the introduction section, it is well established that people tend to misperceive actual levels of economic inequality, as Norton and Ariely (1) originally tested. Following research, however, raised many critical points concerning the method originally used by the authors (for a review on the topic see (2)). For example, Eriksson and Simpson (3) argued that expressing wealth owned by population quintiles in percentages is not only logically demanding, but may also elicit an anchoring effect, where people anchor their responses on an equal distribution (20% per quintile) and then (insufficiently) adjust from there. The authors argued that this method may have led participants to provide responses that reflected a particularly low level of perceived wealth inequality. The authors suggested to solve this issue by changing the linguistic frame. Instead of the original phrasing (i.e., "What percent of the United States’ total wealth is [should be] controlled by the richest 20% of Americans?” (1)), they asked participants for the “average wealth” (i.e., “What is [should be] the average household wealth, in dollars, among the 20% richest households in the United States?” ; (3)). Although this strategy was successful in reducing the underestimation of actual inequality found in Norton and Ariely (1), we argue that this alternative does not fully reflect people’s *lay* perceptions of inequality, given that anchoring effects (for instance, using one’s own wealth as an anchor, or using present inequality levels to estimate past and future inequality) are still likely to manifest. Therefore, in the present study, we pursued a completely different strategy, by providing a multiple-choice format that reduces memory and anchoring biases while, at the same time, reducing cognitive load on respondents.

# **Study 1**

## Additional Measures

In addition to what we reported in the manuscript and for exploratory purposes, two items were used to measure life satisfaction as a control variable (i.e., satisfaction with life in general and satisfaction with the economic situation, both assessed on a Likert scale from 1 = *not at all satisfied* to 5 = *completely satisfied*). We included these items since life satisfaction is negatively affected by levels of wealth inequality of a country (e.g., (4)),

Furthermore, participants were asked how difficult it was for them to make ends meet, on a 5-point Likert scale ranging from 1 (*no difficulty*) to 5 (*a lot of difficulty*)**,** and how frequently they obtained information on the topic of socio-economic inequality in Italy from a set of different media, on a 5-point Likert scale ranging from 1 (*never*) to 5 (*daily*). These sources of information were divided into “conventional” (4 items, e.g., newscast; *α* = .70) and “non-conventional” sources (5 items, e.g., scientific dissemination papers; *α* = .65). Participants were additionally asked to report whether they took interest in rich individuals and/or V.I.P.s, answering on a 5-point Likert scale ranging from 1 (*not at all*) to 5 (*a lot*).

## Causal Attribution Analyses

Past research had linked causal attributions to either status-quo maintenance (5), or to the perception of country-level wealth inequality as just (6).

***Factorial Analysis***

First, we ran a Principal Component Analysis, and identified four factors with eigenvalues greater than 1; as can be seen in S2 Fig the scree-plot also evidenced four relevant factors. As the fourth factor was only comprised of a single item, “Unequal access to education”, which appeared as bifactorial, we then decided to run a Common Factor Analysis with principal axis factoring and varimax rotation, fixing the number of factors to three: 7 items loaded on the first factor, representing individual causes (loadings ranging from .40 to .70), 9 items loaded on the second factor, corresponding to systemic causes (loadings ranging from .38 to .62), and 3 items loaded on the third factor, named criminality causes (loadings ranging from .45 to .83).

*S2 Fig. Scree-plot emerging from (a) Principal Component Analyses (extraction based on eigenvalues); and (b) the Common Factor Analysis with principal axis factoring (varimax rotation)*


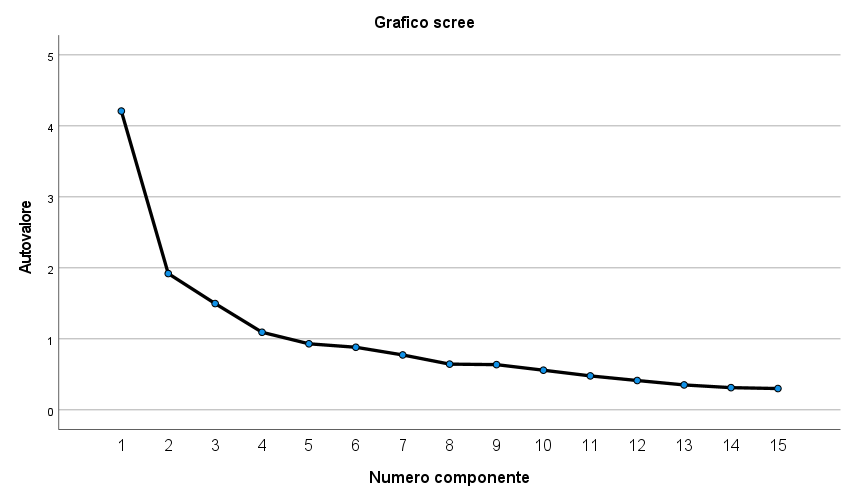


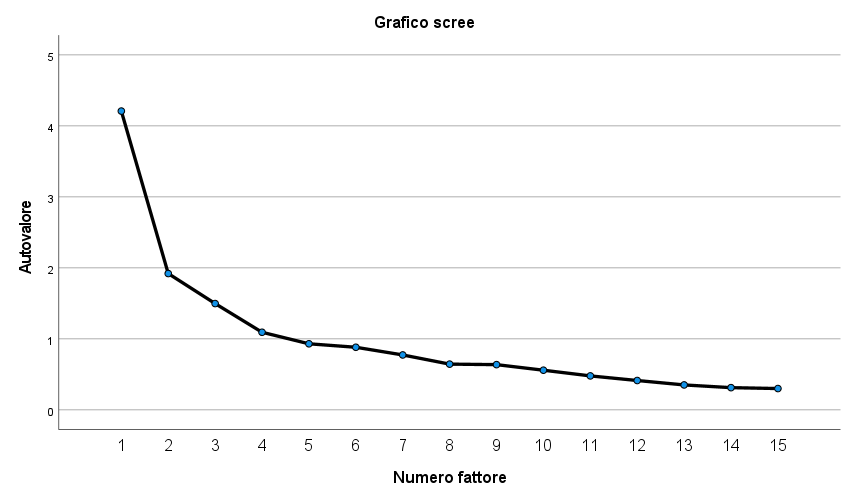


| *S1 Table. Items – 3 Factors Solution (Common Factor Analysis with Principal Axis Factoring – Varimax Rotation) For The Causal Attribution Scale in Study 1.* | | | |
| --- | --- | --- | --- |
| Items | Factor loadings | | |
|  | 1 | 2 | 3 |
| 1. Corruzione [Corruption] | ,01 | ,14 | **,83** |
| 2. Criminalità organizzata [Organized crime] | ,08 | ,05 | **,74** |
| 3. Crisi economica [Economic crisis] | ,07 | **,49** | ,06 |
| 4. Debito pubblico [Public debt] | ,02 | **,58** | ,23 |
| 5. Debolezza del sistema politico [Weakness of the political system] | ,06 | **,47** | ,34 |
| 6. Differenze individuali nei livelli di scolarizzazione  [Individual differences in education levels] | **,53** | ,09 | ,13 |
| 7. Differenze individuali nell'intraprendenza economica  [Individual differences in economic resourcefulness] | **,70** | ,19 | ,03 |
| 8. Differenze individuali nella capacità di gestione del denaro [Individual differences money management] | **,70** | ,12 | ,08 |
| 9. Politiche di genere non egalitarie [Unequal gender policies] | ,28 | **,38** | ,12 |
| 10. Emigrazione [Emigration] | ,24 | **,62** | -,05 |
| 11. Evasione Fiscale [Tax evasion] | ,11 | ,32 | **,45** |
| 12. Immigrazione [Immigration] | ,15 | **,55** | -,03 |
| 13. Iniquo accesso alla scolarizzazione  [Unequal access to education] | **,40** | ,28 | ,07 |
| 14. Mercato del lavoro [job market] | ,27 | **,45** | ,06 |
| 15. Scarsa competenza finanziaria individuale  [Lack of personal financial competence] | **,70** | ,12 | ,08 |
| 16. Scarsa competitività aziendale  [Lack of business competitiveness] | **,60** | ,25 | ,04 |
| 17. Scarso sostegno statale verso le famiglie  [Lack of governmental support for families] | ,22 | **,52** | ,23 |
| 18. Scelte di vita individuali [Personal life choices] | **,64** | ,03 | -,03 |
| 19. Tassazione non equa [Unequal taxation] | ,18 | **,43** | ,23 |
| *Note.* The extraction method was principal axis factoring with an oblique (varimax with Kaiser normalization) rotation. Factor loadings over .40 are in bold. | | | |

Therefore, Internal causes included 7 items: business competitiveness, economic resourcefulness, financial competence, life choices, money management, schooling access, schooling differences. Structural (external) causes included 9 items: economic crisis, emigration, family support, gender policies, immigration, job market, political system, public debt, taxation Criminality (external) causes included 3 items: corruption, criminality, tax evasion

***Exploratory analyses***

To test this exploratory hypothesis, a linear regression model was run, with misperception of wealth inequality (i.e., the difference between actual wealth inequality and estimated present wealth inequality) as dependent variable and internal, structural and criminality causes (all centered) as predictors. The model, however, was non-significant, *R^2^* = .003, *F*(3, 350) = 1.36, *p* = .255.

The same linear regression model was conducted using an index of pessimism for the future (i.e., the difference between estimated future wealth inequality and estimated current wealth inequality) as dependent variable. Criminality positively predicted the pessimism for the future index, *B* = 3.15, 95% CI [.29, 6.01], *β* = .13, *t* = 2.17, *p* = .031, so that the more participants believed that wealth inequality was to be attributed to criminality (external attribution), the more they thought that future inequality would increase above current levels.

Therefore, none of the three types of attributions affected misperception of present wealth inequality. Some interesting results, however, emerged when assessing causal attribution regarding European comparison: the more current inequality was explained by individual causes, the lower Italy was ranked in terms of wealth inequality, while the opposite occurred when it was attributed to structural causes. It appears that causal attributions were linked to general pessimism towards wealth inequality, rather than inequality awareness per se. Consistently, an interesting pattern emerged when considering pessimism towards future inequality: the more people attributed present wealth inequality to criminality-related (external) issues, the more they believed that inequality would rise in the future. As highlighted by national research (Istat, 2017), corruption of the Italian society (particularly when economy is involved) is a particularly worrying systemic issue for Italian citizens, possibly more than other societal issues characteristic of 2016, which could in part explain these specifics findings.

A regression model was conducted with personal, structural, and criminality causes (centered) as predictors and the subjective ranking of Italy as the dependent variable. Structural causes positively predicted the ranking of Italy, *B* = .44, 95% *CI* [.15, .72], *β* = .18, *t* = 2.98, *p* = .003, whereas internal causes predicted it negatively, *B* = -.30, 95% *CI* [-.53, -.06], *β* = -.15, *t* = -2.48, *p* = .014. Therefore, the more participants attributed wealth inequality to structural (external) causes, the higher they thought wealth inequality was in Italy compared to wealth inequality in Italy compared to other countries (i.e., they believed Italy presented higher levels of wealth inequality). To the contrary, the more they attributed inequality to internal causes (such as a people’s poor financial competence), the more optimistic participants were about wealth distribution in Italy compared to other countries.

## European Wealth Distribution Ranking Analyses

A series of one-sample t-tests was conducted to compare the participant’s subjective ranking of each country (i.e., the ranking they assigned to each country, from 1 to 9) with the actual wealth distribution ranking. Results are depicted in S3 Fig, and were all significant (p < .001), with the exception of Norway (p = .799). Participants tended to overestimate wealth inequality in the most equal (i.e., Finland and Denmark), but to underestimate wealth inequality in the most unequal countries (i.e., Spain and UK). Estimates for countries in the middle range were less systematic. Overall, the correlation between mean subjective rankings and actual Gini rankings was r(9) = .71, p = .031, suggesting that participants had a reasonable notion of the relative standing of the 9 countries with respect to wealth inequality.

When ranking Italy in the European wealth distribution task (which was placed 6th out of 9 countries in terms of the actual Gini Index), 11% of participants underestimated Italy’s levels of wealth inequality, 24% correctly guessed the answer, and the remaining 65% overestimated Italy’s levels of wealth inequality. Thus, although participants performed well above chance when ranking the European countries in general, they reliably overestimated wealth inequality in their own country compared to other European countries.

*S3 Fig. European Wealth Distribution Ranking: Comparison of Participant’s Ranking of European Countries with Actual Wealth Distribution Rankings (Study 1)*


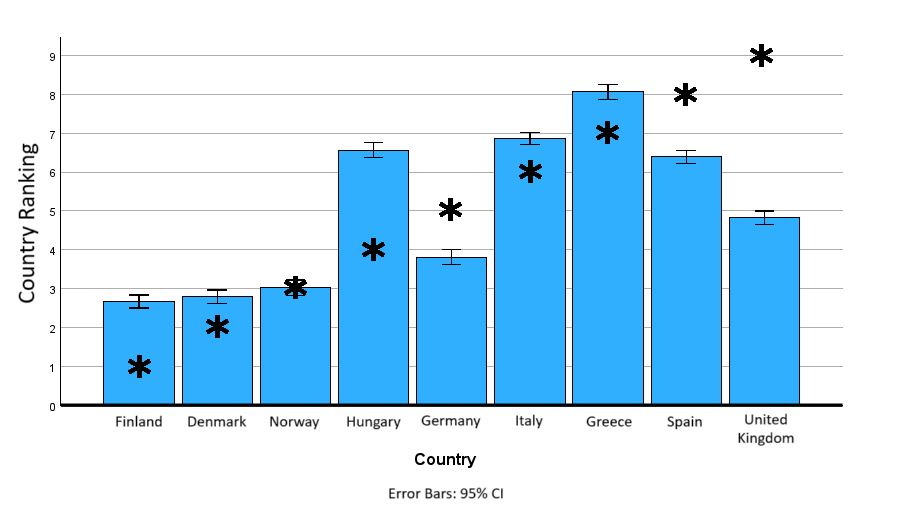


*Note: each asterisk corresponds to the correct ranking of each country based on Gini Index (i.e., from 1 = Finland – lowest wealth inequality; to 9 = UK, highest wealth inequality; see Study 1 – Method “European wealth distribution ranking” section for details on country selection). Comparison was run via t-test*

| S2 Table  *Correlational Tables for Study 1* | | | | | | | | | | | | | | | | | | | | | | | | | | | | | | | | | | | | | | | | |
| --- | --- | --- | --- | --- | --- | --- | --- | --- | --- | --- | --- | --- | --- | --- | --- | --- | --- | --- | --- | --- | --- | --- | --- | --- | --- | --- | --- | --- | --- | --- | --- | --- | --- | --- | --- | --- | --- | --- | --- | --- |
|  | **1** | | **2** | | **3** | | **4** | | **5** | | **6** | | **7** | | **8** | | **9** | | **10** | | **11** | | **12** | | **13** | | **14** | **15** | **16** | **17** | **18** | **19** | **20** | **21** | **22** | **23** | **24** | **25** | **26** | **27** |
| **1. Life Satisfaction** | - | | .37^***^ | | .01 | | .13^*^ | | -.04 | | -.06 | | .06 | | .03 | | -.02 | | .06 | | .03 | | .02 | | .08 | | .02 | -.03 | -.11^*^ | -.06 | .06 | .10 | -.08 | .01 | .01 | -.03 | -.26^***^ | .09 | .04 | .11^*^ |
| **2. Economic Satisfaction** |  | | - | | .16^**^ | | .15^**^ | | -.08 | | -.06 | | .04 | | .01 | | -.07 | | .06 | | -.02 | | -.02 | | -.03 | | -.002 | -.02 | -.001 | -.09 | -.03 | .11^*^ | .07 | .07 | -.09 | -.14^*^ | -.54^***^ | -.01 | -.03 | -.02 |
| **3. Age** |  | |  | | - | | .05 | | -.10 | | .15^**^ | | .01 | | .03 | | .02 | | -.15^**^ | | -.12^*^ | | -.11^*^ | | -.12^*^ | | -.01 | .05 | -.06 | -.02 | .05 | .15^**^ | .07 | -.03 | .02 | -.07 | -.08 | .13^*^ | -.12^*^ | -.09 |
| **4. Monthly Income** |  | |  | |  | | - | | -.05 | | -.08 | | .18^**^ | | .003 | | -.14^*^ | | .08 | | -.10 | | .07 | | .05 | | .07 | .03 | .03 | -.18^**^ | -.13^*^ | -.06 | .10 | .06 | -.01 | -.02 | -.22^***^ | .004 | .06 | .15^**^ |
| **5. Political Orientation** |  | |  | |  | |  | | - | | .05 | | -.07 | | .09 | | .13^*^ | | -.05 | | .10 | | .10 | | .04 | | .01 | .11^*^ | .03 | -.05 | -.05 | -.15^**^ | -.07 | -.06 | -.12^*^ | .04 | .17^*^ | -.18^**^ | -.12^*^ | .01 |
| **6. Present Wealth Inequality** |  | |  | |  | |  | |  | | - | | -.32^***^ | | .27^***^ | | .48^***^ | | -1.0^***^ | | -.45^***^ | | -.03 | | -.15^**^ | | -.02 | -.08 | .04 | .19^***^ | -.05 | .04 | .08 | -.10 | -.07 | -.05 | .04 | -.01 | -.02 | -.05 |
| **7. Ideal Wealth Inequality** |  | |  | |  | |  | |  | |  | | - | | .10 | | -.29^***^ | | .32^***^ | | -.02 | | -.03 | | .004 | | -.02 | .02 | -.03 | .02 | .11^*^ | -.03 | -.04 | .05 | .04 | .01 | -.03 | .09 | .14^**^ | .17^**^ |
| **8. Past Wealth Inequality** |  | |  | |  | |  | |  | |  | |  | | - | | .14^*^ | | -.27^***^ | | -.11 | | .05 | | .002 | | -.03 | -.003 | .01 | .01 | -.02 | .03 | -.05 | .02 | -.01 | -.05 | .04 | .07 | .09 | .10 |
| **9. Future Wealth Inequality** |  | |  | |  | |  | |  | |  | |  | |  | | - | | -.48^***^ | | .57^***^ | | .01 | | -.07 | | .03 | -.07 | .11 | .09 | -.03 | -.12^*^ | .06 | -.003 | .06 | .12^*^ | .09 | .003 | -.05 | .01 |
| **10. Misperception** |  | |  | |  | |  | |  | |  | |  | |  | |  | | - | | .45^***^ | | .03 | | .15^**^ | | .02 | .08 | -.04 | -.19^***^ | .05 | -.04 | -.08 | .10 | .07 | .05 | -.04 | .01 | .02 | .05 |
| **11. Future Pessimism** |  | |  | |  | |  | |  | |  | |  | |  | |  | |  | | - | | .02 | | .08 | | .05 | -.02 | .05 | -.05 | .02 | -.14^*^ | -.03 | .08 | .16^**^ | .17^**^ | .08 | .00 | -.04 | .02 |
| **12. EUR_Finland** |  | |  | |  | |  | |  | |  | |  | |  | |  | |  | |  | | - | | .35^***^ | | .42^***^ | -.21^***^ | -.07 | -.28^***^ | -.42^***^ | -.49^***^ | -.31^***^ | .13^*^ | .04 | -.05 | .12^*^ | .08 | .06 | .13^*^ |
| **13. EUR_Denmark** |  | |  | |  | |  | |  | |  | |  | |  | |  | |  | |  | |  | | - | | .21^***^ | -.23^***^ | -.13^*^ | -.31^***^ | -.29^***^ | -.43^***^ | -.18^**^ | .07 | .07 | -.02 | .08 | .08 | .02 | .18^**^ |
| **14. EUR_Norway** |  | |  | |  | |  | |  | |  | |  | |  | |  | |  | |  | |  | |  | | - | -.20^***^ | -.11^*^ | -.26^***^ | -.42^***^ | -.46^***^ | -.25^***^ | .09 | .15^**^ | .001 | .15^**^ | .02 | .01 | .01 |
| **15. EUR_ Hungary** |  |  | |  | |  | |  | |  | |  | |  | |  | |  | |  | |  | |  | |  | | - | -.25^***^ | -.13^*^ | .07 | .03 | -.18^**^ | .03 | .08 | .02 | -.06 | -.13^*^ | -.08 | -.02 |
| **16. EUR_Germany** |  |  | |  | |  | |  | |  | |  | |  | |  | |  | |  | |  | |  | |  | |  | - | -.20^***^ | -.34^***^ | -.14^**^ | .18^**^ | -.06 | -.15^**^ | -.06 | .02 | -.05 | -.03 | -.03 |
| **17. EUR_Italy** |  |  | |  | |  | |  | |  | |  | |  | |  | |  | |  | |  | |  | |  | |  |  | - | .31^***^ | .12^*^ | -.11^*^ | -.05 | .15^**^ | .12^*^ | .15^**^ | .05 | .17^**^ | -.06 |
| **18. EUR_Greece** |  |  | |  | |  | |  | |  | |  | |  | |  | |  | |  | |  | |  | |  | |  |  |  | - | .32^***^ | -.22^***^ | -.08 | -.04 | -.02 | -.10 | .05 | -.01 | -.02 |
| **19. EUR_Spain** |  |  | |  | |  | |  | |  | |  | |  | |  | |  | |  | |  | |  | |  | |  |  |  |  | - | .10 | -.06 | -.14^**^ | .02 | -.22^***^ | .02 | -.07 | -.08 |
| **20. EUR_UK** |  |  | |  | |  | |  | |  | |  | |  | |  | |  | |  | |  | |  | |  | |  |  |  |  |  | - | -.09 | -.14^**^ | .002 | -.12^*^ | -.09 | -.05 | -.12^*^ |
| **21. Individual Causes** |  |  | |  | |  | |  | |  | |  | |  | |  | |  | |  | |  | |  | |  | |  |  |  |  |  |  | - | .46^***^ | .20^***^ | .05 | .10 | .26^***^ | .07 |
| **22. Structural Causes** |  |  | |  | |  | |  | |  | |  | |  | |  | |  | |  | |  | |  | |  | |  |  |  |  |  |  |  | - | .37^***^ | .23^***^ | .17^**^ | .20^***^ | .06 |
| **23. Criminality Causes** |  |  | |  | |  | |  | |  | |  | |  | |  | |  | |  | |  | |  | |  | |  |  |  |  |  |  |  |  | - | .18^**^ | .07 | .10 | -.02 |
| **24. Economic Hardship** |  |  | |  | |  | |  | |  | |  | |  | |  | |  | |  | |  | |  | |  | |  |  |  |  |  |  |  |  |  | - | .03 | .14^**^ | -.11^*^ |
| **25. Information Sources – Conventional** |  |  | |  | |  | |  | |  | |  | |  | |  | |  | |  | |  | |  | |  | |  |  |  |  |  |  |  |  |  |  | - | .46^***^ | .20^***^ |
| **26. Information Sources – Unconventional** |  |  | |  | |  | |  | |  | |  | |  | |  | |  | |  | |  | |  | |  | |  |  |  |  |  |  |  |  |  |  |  | - | .16^**^ |
| **27. Interest in Rich People and VIPS** |  | |  | |  | |  | |  | |  | |  | |  | |  | |  | |  | |  | |  | |  |  |  |  |  |  |  |  |  |  |  |  |  | - |
| *Note.* EUR = European wealth distribution task.  * p < .05; ** p < .01; *** p < .001 | | | | | | | | | | | | | | | | | | | | | | | | | | | | | | | | | | | | | | | | |

# **Study 2**

| S3 Table  *Correlational Tables for Study 2* | | | | | | | | | | | | | | | | | | | | | | | |
| --- | --- | --- | --- | --- | --- | --- | --- | --- | --- | --- | --- | --- | --- | --- | --- | --- | --- | --- | --- | --- | --- | --- | --- |
|  | **1** | **2** | **3** | **4** | **5** | **6** | **7** | **8** | **9** | **10** | **11** | **12** | **13** | **14** | **15** | **16** | **17** | **18** | **19** | **20** | **21** | **22** | **23** |
| **1. Life Satisfaction** | 1 | .55^***^ | .04 | -.02 | -.14^*^ | .03 | .04 | -.05 | -.08 | -.03 | -.11 | .05 | .04 | -.04 | .04 | .11 | -.13^*^ | -.08 | .07 | -.06 | .13^*^ | -.10 | .02 |
| **2. Economic Satisfaction** |  | 1 |  | .05 | -.05 | .05 | -.01 | .01 | .07 | -.05 | .02 | -.07 | -.02 | -.07 | .11 | .06 | -.13^*^ | -.07 | .06 | .13^*^ | .06 | -.10 | -.05 |
| **3. Age** |  |  | 1 | -.01 | -.16^*^ | .04 | -.02 | -.04 | -.01 | -.04 | -.03 | -.06 | -.05 | -.10 | .06 | .08 | .03 | .03 | .05 | -.02 | .02 | .12 | .00 |
| **4. Monthly Income** |  |  |  | 1 | -.05 | .04 | -.04 | -.04 | .03 | -.04 | .002 | -.06 | -.06 | .01 | .02 | .03 | .001 | .06 | -.02 | -.01 | -.05 | .00 | .03 |
| **5. Political Orientation** |  |  |  |  | 1 | .15^*^ | -.14^*^ | .24^***^ | .21^**^ | -.15^*^ | .08 | -.15^*^ | -.01 | .05 | .08 | .06 | -.15^*^ | -.04 | -.01 | .12 | -.08 | .02 | .09 |
| **6. Present Wealth Inequality** |  |  |  |  |  | 1 | -.15^*^ | .50^***^ | .49^***^ | -1.0^***^ | -.40^***^ | .02 | -.06 | -.08 | .11 | .06 | -.02 | -.08 | -.07 | .10 | -.06 | .02 | .05 |
| **7. Ideal Wealth Inequality** |  |  |  |  |  |  | 1 | -.15^*^ | -.10 | .15^*^ | .06 | .20^**^ | .03 | .13^*^ | -.14^*^ | -.05 | .002 | -.03 | .03 | -.13^*^ | .01 | -.10 | -.08 |
| **8. Past Wealth Inequality** |  |  |  |  |  |  |  | 1 | .61^***^ | -.50^***^ | .18^**^ | -.04 | .01 | -.08 | .07 | -.06 | .09 | -.004 | -.01 | .03 | -.08 | .03 | .13 |
| **9. Future Wealth Inequality** |  |  |  |  |  |  |  |  | 1 | -.49^***^ | .60^***^ | .02 | .03 | -.07 | -.002 | .04 | .10 | -.12 | -.08 | .11 | -.16^*^ | .04 | .03 |
| **10. Misperception of Wealth Inequality** |  |  |  |  |  |  |  |  |  | 1 | .40^***^ | -.02 | .06 | .08 | -.11 | -.06 | .02 | .08 | .07 | -.10 | .06 | -.02 | -.05 |
| **11. Future Pessimism** |  |  |  |  |  |  |  |  |  |  | 1 | .01 | .09 | .00 | -.08 | -.04 | .10 | -.05 | .01 | .01 | -.11 | .02 | -.02 |
| **12. EUR_Finland** |  |  |  |  |  |  |  |  |  |  |  | 1 | .26^***^ | .39^***^ | -.14^*^ | -.19^**^ | -.21^**^ | -.46^***^ | -.38^***^ | -.23^***^ | .09 | -.09 | .04 |
| **13. EUR_Denmark** |  |  |  |  |  |  |  |  |  |  |  |  | 1 | .17^**^ | -.26^***^ | -.18^**^ | -.15^*^ | -.34^***^ | -.29^***^ | -.15^*^ | -.01 | .06 | .06 |
| **14. EUR_Norway** |  |  |  |  |  |  |  |  |  |  |  |  |  | 1 | -.18^**^ | -.24^***^ | -.33^***^ | -.51^***^ | -.31^***^ | -.08 | .02 | .02 | .07 |
| **15. EUR_ Hungary** |  |  |  |  |  |  |  |  |  |  |  |  |  |  | 1 | -.27^***^ | -.29^***^ | .09 | .04 | -.23^***^ | -.01 | -.08 | -.03 |
| **16. EUR_Germany** |  |  |  |  |  |  |  |  |  |  |  |  |  |  |  | 1 | -.002 | -.06 | -.07 | .17^**^ | .09 | .05 | .02 |
| **17. EUR_Italy** |  |  |  |  |  |  |  |  |  |  |  |  |  |  |  |  | 1 | .25^***^ | .02 | -.13^*^ | .03 | .17^**^ | .04 |
| **18. EUR_Greece** |  |  |  |  |  |  |  |  |  |  |  |  |  |  |  |  |  |  | .192^*^ | -.26^***^ | -.04 | .05 | -.05 |
| **19. EUR_Spain** |  |  |  |  |  |  |  |  |  |  |  |  |  |  |  |  |  |  | 1 | -.09 | -.04 | -.03 | -.02 |
| **20. EUR_UK** |  |  |  |  |  |  |  |  |  |  |  |  |  |  |  |  |  |  |  |  | -.11 | -.13^*^ | -.12 |
| **21. Individual Causes** |  |  |  |  |  |  |  |  |  |  |  |  |  |  |  |  |  |  |  |  | 1 | .33^***^ | .17^**^ |
| **22. Structural Causes** |  |  |  |  |  |  |  |  |  |  |  |  |  |  |  |  |  |  |  |  |  | 1 | .34^***^ |
| **23. Criminality Causes** |  |  |  |  |  |  |  |  |  |  |  |  |  |  |  |  |  |  |  |  |  |  | 1 |
|  | | | | | | | | | | | | | | | | | | | | | | | |
| *Note.* All *N*s = 246, except for: Past Wealth Inequality (*N* = 242), Future Wealth Inequality and Future Pessimism (*N* = 236). EUR = European wealth distribution task.  * p < .05; ** p < .01; *** p < .001 | | | | | | | | | | | | | | | | | | | | | | | |

## Causal Attribution Analyses

A linear regression model was conducted using misperception of wealth inequality (i.e., the difference between actual wealth inequality and estimated present wealth inequality) as dependent variable and internal, structural, and criminality causes (all centered) as predictors. As for Study 1, the model was non-significant, *R*^2^ = -.005, *F*(3, 242) = .58, *p* = .631. The same linear regression model was conducted using the index of pessimism for the future (i.e., the difference between estimated future wealth inequality and estimated present wealth inequality) as dependent variable. Differently from Study 1, however, the model was non-significant, *R^2^* = .001, *F* (3, 229) = 1.07, *p* = .362.

A regression model was conducted using internal, structural, and criminality causes (centered) as a predictors and Italy ranking as dependent variable. Structural causes positively predicted Italy ranking, B = .44, 95% CI [.10, .78], β = .18, t = 2.56, p = .011. The more participants attributed wealth inequality to structural (external) causes, the more they were pessimistic about levels of wealth inequality in Italy compared to other countries (i.e., they believed Italy presented higher wealth inequality).

Again, causal attributions were unrelated to misperception of present wealth inequality. Structural (but not internal) causes remained a stable predictor of the perceived rank of Italy compared to other European countries, but did not predict the estimates of future wealth distribution in Italy. A possible reason for this difference compared to the 2016 data may lie in the fact that Italians in 2021 were more concerned with other issues, in particular those related to the COVID-19-pandemic.

The only causal attribution that was predictive of future (vs. present) wealth inequality was criminality, but only in Study 1. The more participants blamed current inequality on criminality, the more they thought wealth distribution would become even more unequal in the future. The difference between our findings and those of prior studies may be explained by the fact that our causal attribution measure was developed to evaluate participants’ explanations of wealth inequality, rather than poverty: the underlying causes of wealth inequality may be less clear to laypeople.

## European Wealth Distribution Ranking Analyses

A series of one-sample t-tests was conducted to compare the participant’s subjective ranking of each country (i.e., the ranking they assigned to each country, from 1 to 9) with the actual wealth distribution ranking. Results are depicted in S4 Fig, and were all significant (p < .001). As in Study 1, participants tended to overestimate wealth inequality in the most equal countries, while conversely underestimating wealth inequality in the most unequal countries. Differently from Study 1, estimations are much more polarized: participants assumed Hungary to be a much more unequal country than it actually is, they slightly overestimated inequality levels in Greece, and slightly underestimated inequality levels in Italy and in the UK. Overall, the correlation between mean subjective rankings and actual Gini rankings was r(9) = .50, p = .174^[[1]](#footnote-1)^, suggesting that participants had a reasonable notion of the relative standing of the 9 countries with respect to wealth inequality.

When ranking Italy in the European wealth distribution task (which was placed 8th out of 9 countries in terms of the actual Gini Index), 79% of participants underestimated Italy’s levels of wealth inequality, 17% correctly guessed the answer, and the remaining 4% overestimated Italy’s levels of wealth inequality. Thus, although participants performed well above chance when ranking the European countries in general, they reliably underestimated wealth inequality in their own country compared to other European countries.

*S4 Fig. European Wealth Distribution Ranking: Comparison of Participant’s Ranking of European Countries with Actual Wealth Distribution Rankings (Study 2)*


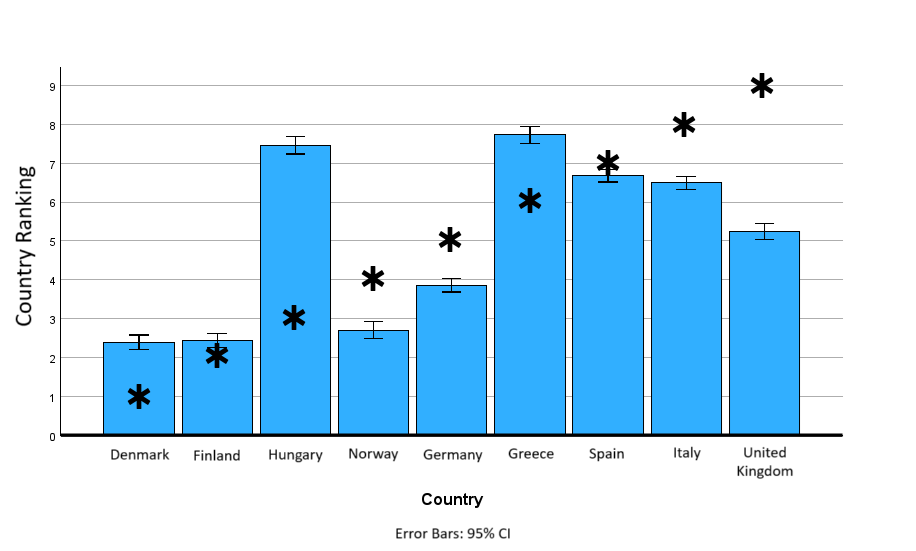


*Note: each asterisk corresponds to the correct ranking of each country based on Gini Index (i.e., from 1 = Denmark – lowest wealth inequality; to 9 = UK, highest wealth inequality; see Study 2 – Method “European wealth distribution ranking” section for details on country selection). Comparison was run via t-test*

## Analyses excluding one participant for completion times

***Inequality Estimates***

**Estimated and Ideal Inequality.** Hypothesis 1a could not be tested, as the correct option for current inequality in 2021 was not available to participants. In support of Hypothesis 1b, participant’s ideal wealth distribution (*M* = 30.59, *SD* = 14.71) was lower than their own estimates (*M* = 49.07, *SD* = 16.01), paired-sample *t*(244) = 12.25, *p* = .010. At the same time, in line with Hypothesis 1b, the ideal distribution exceeded a perfectly equal distribution (20%), one-sample *t*(244) = 11.27, *p* < .001, suggesting that participants desired some degree of inequality.

**Comparing Past, Present, and Future Inequality.** A repeated measures ANOVA with Greenhouse-Geisser correction showed a difference among the three estimation variables between each other, *F*(1, 231) = 9.65, *p* < .001, *η^2^_p_* = .04. Estimation of present inequality scores (*M* = 49.08, *SD =* 15.82) were found to be significantly lower than estimation of future inequality ones (*M* = 52.25, *SD =* 18.05), *t*(231) = -2.83, *p* = .005. Estimation of present inequality scores however were not higher than estimation of past inequality ones (*M* = 47.81, *SD =* 15.20), *t*(231) = 1.25, *p* = .213.

***Comparing 2016 With 2021 Data***

Comparing expectations for 2026, namely estimated for 10 years in the future for Study 1 (*M* = 51.60, *SD* = 18.87) and 5 years in the future for Study 2 (*M* = 52.25, *SD* = 18.05) paired-samples t-tests revealed no differences, *t*(553) = -.41, *p* = .680. Similarly, comparing estimates for the present in 2016 (*M* = 46.47, *SD* = 17.37) with estimates 5 years in the past in 2021 (*M* = 47.81, *SD* = 15.20) showed again no differences, *t*(553) = -.94, *p* = .345, indicating that participants in the first study and participants in the second study estimated on average similar scores for set time stamps.

***European Wealth Distribution Ranking***

We ran a regression model with systemic, individual, and criminality causes as a predictor and Italy ranking as a dependent variable. Systemic causes positively predicted Italy ranking, *B* = .44, 95% CI [.10, .78], *β* = .18, *t* = 2.55, *p* = .012. In other words, the more participants attributed inequality to the system, the more they were pessimistic about inequality in Italy compared to other countries (i.e., they believed Italy presented higher wealth inequality).

A regression model was conducted using internal, structural, and criminality causes (centered) as a predictors and Italy ranking as dependent variable. Structural causes positively predicted Italy ranking, B = .44, 95% CI [.10, .78], β = .18, t = 2.56, p = .011. The more participants attributed wealth inequality to structural (external) causes, the more they were pessimistic about levels of wealth inequality in Italy compared to other countries (i.e., they believed Italy presented higher wealth inequality).

# **References**

1. Norton MI, Ariely D. Building a Better America—One Wealth Quintile at a Time. Perspect Psychol Sci. 2011 Jan;6(1):9–12.

2. Marandola G, Xu Y. (Mis-) Perception of Inequality: Measures, Determinants, and Consequences [Internet]. Rochester, NY; 2021 [cited 2022 Jun 22]. Available from: https://papers.ssrn.com/abstract=3898673

3. Eriksson K, Simpson B. What do Americans know about inequality? It depends on how you ask them. Judgm Decis Mak. 2012 Nov;7(6):741–5.

4. Zaborskis A, Grincaite M, Lenzi M, Tesler R, Moreno-Maldonado C, Mazur J. Social Inequality in Adolescent Life Satisfaction: Comparison of Measure Approaches and Correlation with Macro-level Indices in 41 Countries. Soc Indic Res. 2019 Feb 1;141(3):1055–79.

5. Rodriguez-Bailon R, Bratanova B, Willis GB, Lopez-Rodriguez L, Sturrock A, Loughnan S. Social Class and Ideologies of Inequality: How They Uphold Unequal Societies: Social Class and Ideologies of Inequality. J Soc Issues. 2017 Mar;73(1):99–116.

6. Schneider SM, Castillo JC. Poverty Attributions and the Perceived Justice of Income Inequality: A Comparison of East and West Germany. Soc Psychol Q. 2015 Sep;78(3):263–82.

1. We interpret the correlation regardless of the *p* value, given the low number of units (*N* = 9) [↑](#footnote-ref-1)
